# Adolescent Toluene Inhalation in Rats Affects White Matter Maturation with the Potential for Recovery Following Abstinence

**DOI:** 10.1371/journal.pone.0044790

**Published:** 2012-09-18

**Authors:** Jhodie Rubina Duncan, Alec Lindsay Ward Dick, Gary Egan, Scott Kolbe, Maria Gavrilescu, David Wright, Dan Ian Lubman, Andrew John Lawrence

**Affiliations:** 1 Division of Behavioural Neuroscience, Florey Neuroscience Institutes, University of Melbourne, Melbourne, Victoria, Australia; 2 Department Anatomy & Neuroscience, University of Melbourne, Melbourne, Victoria, Australia; 3 Centre for Neuroscience Research, University of Melbourne, Melbourne, Victoria, Australia; 4 Turning Point Alcohol & Drug Centre, Eastern Health and Monash University, Melbourne, Victoria, Australia; Radboud University, The Netherlands

## Abstract

Inhalant misuse is common during adolescence, with ongoing chronic misuse associated with neurobiological and cognitive abnormalities. While human imaging studies consistently report white matter abnormalities among long-term inhalant users, longitudinal studies have been lacking with limited data available regarding the progressive nature of such abnormalities, including the potential for recovery following periods of sustained abstinence. We exposed adolescent male Wistar rats (postnatal day 27) to chronic intermittent inhaled toluene (3,000 ppm) for 1 hour/day, 3 times/week for 8 weeks to model abuse patterns observed in adolescent and young adult human users. This dosing regimen resulted in a significant retardation in weight gain during the exposure period (p<0.05). In parallel, we performed longitudinal magnetic resonance imaging (T_2_-weighted) and diffusion tensor imaging prior to exposure, and after 4 and 8 weeks, to examine the integrity of white matter tracts, including the anterior commissure and corpus callosum. We also conducted imaging after 8 weeks of abstinence to assess for potential recovery. Chronic intermittent toluene exposure during adolescence and early adulthood resulted in white matter abnormalities, including a decrease in axial (p<0.05) and radial (p<0.05) diffusivity. These abnormalities appeared region-specific, occurring in the anterior commissure but not the corpus callosum and were not present until after at least 4 weeks of exposure. Toluene-induced effects on both body weight and white matter parameters recovered following abstinence. Behaviourally, we observed a progressive decrease in rearing activity following toluene exposure but no difference in motor function, suggesting cognitive function may be more sensitive to the effects of toluene. Furthermore, deficits in rearing were present by 4 weeks suggesting that toluene may affect behaviour prior to detectable white matter abnormalities. Consequently, exposure to inhalants that contain toluene during adolescence and early adulthood appear to differentially affect white matter maturation and behavioural outcomes, although recovery can occur following abstinence.

## Introduction

The purposeful abuse of inhaled chemical vapours to produce self-intoxication and/or altered mental state is a significant public health concern, especially among adolescents and young adults. Experimentation with inhalants occurs earlier than with most other drugs of abuse as they are cheap, legal, readily accessible and provide a rapid ‘high’ [1]. Epidemiological studies suggest adolescents and young adults who continue to abuse inhalants chronically have higher levels of anxiety, depressive symptoms, impulsivity and antisocial behaviour [2] with many experiencing major psychiatric problems and/or progressing to illicit drug use [1]–[3].

The deleterious impact of inhalant abuse during adolescence and early adulthood is hypothesized to relate, in part, to an increased vulnerability to injury and/or long-term neuroadaptations as a result of the substantial brain maturation that is occurring during this period [4]. Indeed, animal studies suggest that adolescents display different sensitivity to the acute effects of inhalants [5], altered locomotor activity during recovery [6] and different neurochemical changes in the frontal cortex and striatum [7], when compared to adult animals. However, the long-term consequences of these different sensitivities are poorly understood.

The volatile solvent toluene (methyl benzene) is found in many household products including paints, thinners and aerosols and has high potential for abuse due to its ability to modulate neural reward pathways [8], [9]. Toluene is also believed to be associated with the acute and chronic toxic effects following abuse of inhalants, including brain injury. Human imaging studies report predominantly white matter lesions among chronic inhalant abusers [10], which correlate with cognitive and behavioural deficits such as decreased IQ, tremors, language and memory difficulties [11], [12]. Furthermore, both the extent and type of white matter abnormalities observed in inhalant abusers correlate with severity of cognitive impairments [13] and duration of abuse [14]. However, few studies have specifically investigated recovery of function [15]–[18] or resolution of white matter injury [15], [17], [19] after prolonged exposure to inhalants has ceased.

In this study we aimed to quantify longitudinal behavioural and white matter changes in an adolescent rat model of chronic intermittent toluene (CIT) exposure, which has not been previously explored. As there is no one discrete behavioural phenotype associated with white matter deficits, we chose to assess locomotor activity (including horizontal and vertical plane activity) and motor coordination. This rationale was based on the fact that 1) locomotor activity is sensitive to most changes in behaviour and is transiently affected following acute exposure to toluene [9], 2) these parameters are often used to assess the degree of demyelination in the corpus callosum following exposure to cuprizone in mice [20], and 3) altered diffusion tensor imaging (DTI) parameters in the corpus callosum have recently been shown to correlate with poor motor performance in patients suffering from stroke injury [21]. Concomitantly, we characterised white matter changes in both the anterior commissure and the corpus callosum using MRI and DTI as sensitive, reliable and non-invasive techniques. DTI was chosen as it uses the anisotropic diffusion properties of water molecules (quantity and direction) to characterize microstructural changes of white matter pathways, and as such pathological processes which affect the barriers of diffusion of water molecules will alter DTI signals [22]. The DTI parameters investigated in this study included fractional anisotropy (FA), axial diffusivity (D_A_), radial diffusivity (D_R_) and mean diffusivity (D_M_). FA relates to the degree to which diffusion of water molecules is directionally dependent and provides overall information about white matter pathologies but has no clear relationship to a single pathological change. Conversely, D_A_ relates to the diffusion of water molecules parallel to fibers and is believed to reflect information about axons [23]. D_R_ relates to the diffusion of water molecules perpendicular to fibers and is believed to reflect information about myelination while mean diffusivity (D_M_) reflects a combination of both D_A_ and D_R_ [22], [24]. However, it is over-simplified to make direct associations between white matter structures and DTI parameters following insults [25]. We hypothesized that CIT inhalation during adolescence and early adulthood would result in white matter abnormalities in the maturing brain and that this would be associated with altered behaviour, specifically impaired locomotor activity and coordination. We assessed both the anterior commissure and the corpus callosum to investigate whether region-specific vulnerabilities to CIT may exist. We also investigated whether MRI and/or DTI detectable white matter abnormalities showed potential for recovery following a period of sustained abstinence.

## Materials and Methods

### Ethics

All experiments were performed in accordance with the Prevention of Cruelty to Animals Act, 1986 under the guidelines of the Australian National Health and Medical Research Council Code of Practice for the Care and Use of Animals for Experimental Purposes in Australia. The protocol was approved by the Florey Neuroscience Institutes animal ethics committee (09-101) and experiments were designed to result in minimal suffering to the animals.

### Animals

Adolescent male Wistar rats (∼postnatal day (PND) 24) were obtained from the Australian resources centre (Perth, Australia). Adolescence in rats ranges from weaning at PND 21 to adulthood at PND 60 [26]–[28]. Rats were pair housed, maintained on a 12-hour light/dark cycle (lights on at 0700) and given access to food and water *ad libitum*. Rats were acclimatised for 3 days prior to any experimental manipulation.

### Toluene inhalation exposure

Rats were acclimatised to the laboratory at least 1 hour prior to exposure to toluene or air, during which time their body weights were recorded. Exposure to vapourised toluene was conducted in specialised chambers constructed using toluene resistant materials (Alternative Plastics Pty. Limited, North Melbourne, Vic, Australia) and fittings (Swagelok, Broadmeadows, Vic, Australia) as previously described [29]. Briefly, each single unit (36.6 cm wide×19.5 cm high×17.2 cm deep) housed two chambers (17.6 cm wide×16.5 cm high×16.4 cm deep). Chambers were connected to a toluene vapour system whereby air was pumped through liquid toluene (1.08389, purity >99.8%, Merck, Vic, Australia) in a gas wash-bottle to produce toluene vapour. Toluene, air and mixed gas flow meters allowed the regulation of the desired concentration of toluene vapour in the exposure chambers. The concentration of toluene, both prior to and during an exposure session, in each chamber was verified using an inline gas chromatography system (Shimadzu Corporation, Kyoto, Japan) calibrated utilising toluene of known concentrations (BOC, Vic., Australia). Pilot studies indicated deviations from opening the lid to place the rat in the chamber were restored within 3 minutes. A minimum of 3 readings were taken per session with deviations greater than 100 ppm of the desired toluene concentration being corrected by altering the vapourisation of liquid toluene. Chambers of similar design but exposed to room air only were utilised for control animals (0 ppm exposure).

Rats were randomly assigned to receive either air or CIT (3,000 ppm) whereby either room air or toluene was inhaled for 1 hr per day, 3 days per week (Monday, Wednesday, Friday), for 4 weeks (n = 12 per group for basal locomotor activity, n = 10 per group for organ weights), 8 weeks (n = 6 per group for basal locomotor activity and rotarod, n = 10 per group for body and organ weights) or 8 weeks followed by 8 weeks abstinence (n = 6 per group for MRI and DTI). Consequently, 4 and 8 week exposure periods took animals from early to late adolescence (∼PND 55) or early adulthood (∼PND 80), respectively (28). This exposure paradigm was employed to mirror the human pattern of toluene abuse [30]. The concentration employed elicits a conditioned place preference in rodents [29], [31], an index of positive reinforcement, as well as acutely altering dopamine release in brain regions associated with drug reward [8]. After 1 hour of exposure, rats were placed back into their home cages and semi-isolated from other rats for at least 1 hour to avoid the possible confounds of olfactory stimulation by toluene scent on the fur. All chambers were briefly cleaned with 70% ethanol between sessions. Exposures were conducted at room temperature (∼21°C) under normal lighting and each rat was exposed at approximately the same time each day.

### Basal locomotor activity

Basal locomotor activity was recorded 3 days after 4 (n = 12 per group) and 8 (n = 6 per group) weeks exposure to air or CIT. Rats were habituated to the locomotor cell room for at least 12 hours and sessions were run in the mornings (light cycle) but at a low light setting. Rats were placed in the centre of locomotor arenas (41 cm wide×41 cm deep×41 cm high), encased by two photobeam rings (TruScanTM Photobeam; Coulbourn Instruments, Allentown, PA, USA) 4 cm and 17 cm high. Horizontal and vertical plane movements were recorded in a single test session lasting for 15 minutes.

### Rotarod

Motor co-ordination was tested using a rotarod (Ugo Basile, Italy) 8 days after 8 weeks exposure to air or CIT (n = 6 per group). Rats underwent two training sessions, one at a fixed speed of 4 rpm and one accelerating from 4 rpm to 24 rpm, separated by 15 minutes. During this time, rats were required to remain on the rod for two minutes - if animals fell during this time they were returned to the rod. Four hours after training, rats were subjected to three 5 minute test sessions, each 15 minutes apart. Rats were placed on the rod, which accelerated from 4 rpm to 24 rpm, and the latency to fall was recorded. Motor co-ordination was quantified as time spent on the rotarod prior to falling.

### Organ weights

Following 4 and 8 weeks exposure (n = 10 per group), rats were weighed and then euthanized via an overdose of Lethabarb (1 ml/kg, i.p). The brain and lungs (primary site of exchange for inhaled toluene) were dissected and weighed.

### Imaging

Imaging scans in the same air (n = 6) or CIT-exposed (n = 6) rats were undertaken prior to exposure (baseline, 0 weeks), after 4 and 8 weeks exposure and following 8 weeks abstinence (16 weeks) using a Bruker 4.7 Tesla small animal MRI scanner. It should be noted that due to the limitations of the size of the surface receiver coil proportional to the size of rats at the 16 week time point, these final scans were performed *ex-vivo*. For the 0, 4 and 8 week scans, rats were initially anaesthetised with 4% isoflurane mixed with oxygen and medical air, placed in the prone position on a purpose-built MRI compatible animal holder, and the head fixed in position with ear and tooth bars. Anaesthesia was maintained using a nose cone delivering 1.5–2.5% isoflurane mixed with oxygen and medical air. The animal's respiration was continuously monitored throughout the experiment using a pressure sensitive pad (Small Animal Instruments Inc., New York, USA) positioned under the diaphragm. A decoupled surface receiver coil (Rapid Biomedical GmbH, Rimpar, Germany) was placed over the head. Imaging consisted of a 3-plane localizer sequence, a multi-echo T_2_-weighted sequence and a diffusion-tensor sequence. The total scanning time was approximately 2 hours per animal. Scans were not performed on the same days as exposure to CIT.

For scans conducted at 16 weeks, the rats were euthanized via an overdose of Lethabarb (1 ml/kg, i.p) and were transcardially perfused with phosphate buffered saline (0.1 M, pH 7.4) followed by 4% paraformaldehyde in phosphate buffered saline. Brains were weighed then postfixed overnight in 4% paraformaldehyde and 10% sucrose at 4°C. Brains were washed for 2×30 minutes in phosphate buffered saline and embedded in 2% agar before being scanned. Lungs from these animals were also dissected and weighed.

### MRI

**Figure 1:**
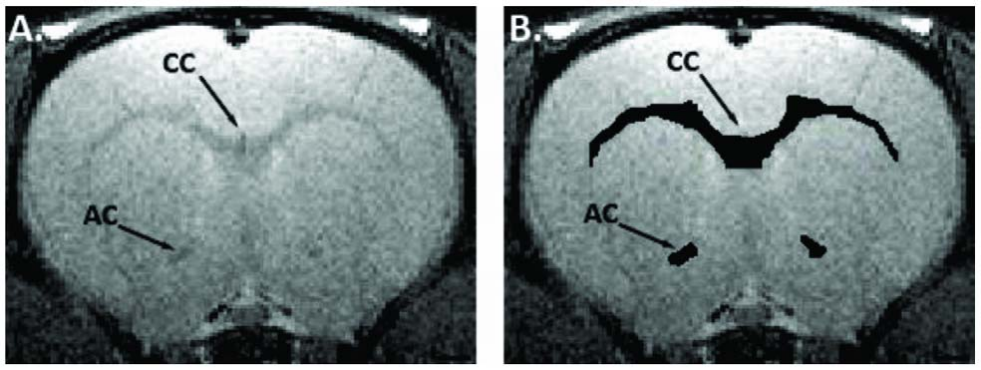
Regions of interest from MRI scans. Representative T_2_-weighted MR images from the rat forebrain (A) and delineation of the regions of interest (B, black) included in volumetric and DTI data analysis. (AC) Anterior commissure; (CC) Corpus callosum. Scale bar = 1 mm.

T_2_ images were acquired using a multi-slice, multi-echo sequence with the following imaging parameters: repetition time = 3,000 ms, 16 echoes with echo times (TE) = 11, 22, etc and 176 ms, matrix size = 192×192, field of view = 2.5×2.5 cm^2^, resolution = 130×130 µm^2^, 15 slices, slice thickness = 1 mm, number of averages = 3, total time ∼25 min. On coronal T_2_ images, averaged across TE to reduce noise, regions of interest including the anterior commissure (bregma 3.2 to −0.4, 3–4 sections per animal) and the corpus callosum (bregma 1.6 to −5.3, genu: 1 section, body: 4–6 sections and splenium: 1 section per animal) in accordance to the rat atlas [32] were delineated based on the voxel intensity and the volume (mm^3^) determined using the FSL software suite (www.fmrib.ox.ac.uk/fsl) (Figure 1). In the anterior commissure, measurements were performed in each hemisphere and combined to give a mean value for each rat. We restricted our analysis to the anterior commissure and corpus callosum as they represent two large discrete white matter pathways in the rodent brain. Furthermore, the genu, body and splenium of the corpus callosum were analysed separately due to the reported alterations in FA values in these regions during maturation [33], attributed to microstructural differences especially with reference to fibre density, axonal diameter [34] and myelin content that are thought to reflect functional specialization.

### DTI

Images were acquired using a spin-echo echo-planar imaging sequence with the following parameters: repetition time = 3,000 ms, TE = 74.35 ms, matrix size = 256×256, field of view = 3.32×3.32 cm^2^, resolution = 130×130 µm^2^, 15 slices, slice thickness = 1 mm and number of repetitions = 3. One B-zero image and 30 non-collinear diffusion directions were acquired with diffusion duration (δ) = 4 ms, diffusion separation (Δ) = 20 ms and diffusion weighting b = 827.5 s/mm^2^, total duration ∼35 min. As per the T_2_ images (Figure 1) regions of interest including the anterior commissure (bregma 3.2 to −0.4, 3–4 coronal sections per animal) and the corpus callosum (bregma 1.6 to −5.3, genu: 1 section, body: 4–6 sections and splenium: 1 section per animal) in accordance to the rat atlas [32] were delineated based on the voxel intensity from B-zero images. In the anterior commissure, measurements were performed in each hemisphere and combined to give a mean value for each rat. DTI parameters were calculated using the FSL software suite (www.fmrib.ox.ac.uk/fsl) and included FA (scale 0–1), D_A_ (mm^2^/sec), D_R_ (mm^2^/sec) and D_M_ (mm^2^/sec). As fixation may affect DTI parameters [22], data from *ex vivo* scans at 16 weeks were analysed independently from *in vivo* data from 0–8 week scans and treatment affects were compared only within this group and not across time points.

### Statistical analysis

Body weights, basal locomotor activity (5 min time bins), rotarod and imaging data (0–8 weeks) were analysed using a 2-way repeated-measures (RM) analysis of variance (ANOVA) with time and treatment as factors. Post-hoc analysis was undertaken using the Holm-Sidak all pairwise multiple comparisons. Body weights after 8 weeks exposure, organ weights, basal locomotor activity (totals) and imaging data collected *ex vivo* (16 weeks) were analysed using a student's *t*-test. All statistical analysis was performed using SigmaStat 3.5 (Jandel, San Jose, CA, USA). Graphs were produced using GraphPad Prism 5 (GraphPad Software, San Diego, CA, USA). Data are presented as mean or mean of means (imaging data only) ± SEM. In all analyses statistical significance was accepted at *p*≤0.05.

## Results

### Effects of CIT exposure on growth

**Figure 2:**
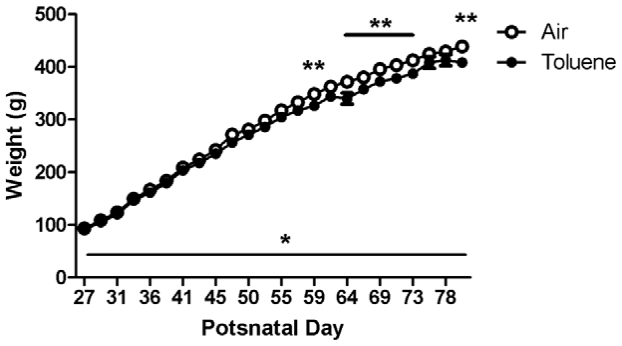
Body weights during the exposure period. Body weight (g) throughout the 8 week exposure period to chronic intermittent toluene or air beginning postnatal day 27. Data are expressed as mean (± SEM), n = 10 per group. *p<0.05 main effect of time, **p<0.05 difference between groups (2-way RM ANOVA with Holm–Sidak post-hoc tests).

There was no difference between CIT and air-exposed rats in body, brain or lung weight after 4 weeks exposure (Table 1). There was however, a main effect of time on body weight (F_(23,414)_ = 2406.838, p<0.001) but not treatment, as well as a significant interaction (time×treatment) between these factors (F_(23,414)_ = 4.473, p<0.001) throughout the 8 week exposure period (PND 27-80, 2-way RM ANOVA). Subsequent post-hoc analysis revealed that exposure to CIT during adolescence and early adulthood resulted in a small but significant retardation on increased body weight, which was evident from PND 59 onwards, over 5 weeks after the initiation of exposure (Figure 2). The effects of CIT on body weight following 8 weeks exposure were still evident after 12 days of abstinence (*t-test*, p = 0.012), but this was not associated with altered brain or lung weight when compared to controls (Table 1). Following 8 weeks of abstinence, there were no statistical differences on any parameter measured between groups (Table 1).

**Table 1:** Body and organ weights during the 8 week exposure period and following 8 weeks Abstinence.

	4 Weeks Exposure: 72 hr Recovery		8 Weeks Exposure: 12 Days Recovery		8 Weeks Exposure: 8 Weeks Recovery	
Weight	Air (10)	CIT (10)	Air (10)	CIT (10)	Air (6)	CIT (6)
Body (g)	302±12	296±8	481±6	448±10*	482±7	499±19
Brain (g)	1.42±0.02	1.45±0.03	2.03±0.04	2.00±0.03	1.85±0.07	1.89±0.05
Lung (g)	1.85±0.16	2.05±0.31	2.27±0.25	1.82±0.08	2.24±0.37	1.73±0.14

Note: Weights after 8 weeks exposure include 12 days of recovery. Data are expressed as mean (± SEM).

* *p*<0.05 compared to air-exposed animals at 12 days recovery (*t*-test). CIT: chronic intermittent toluene.

### Effect of CIT exposure on basal locomotor and rotarod activity

**Figure 3:**
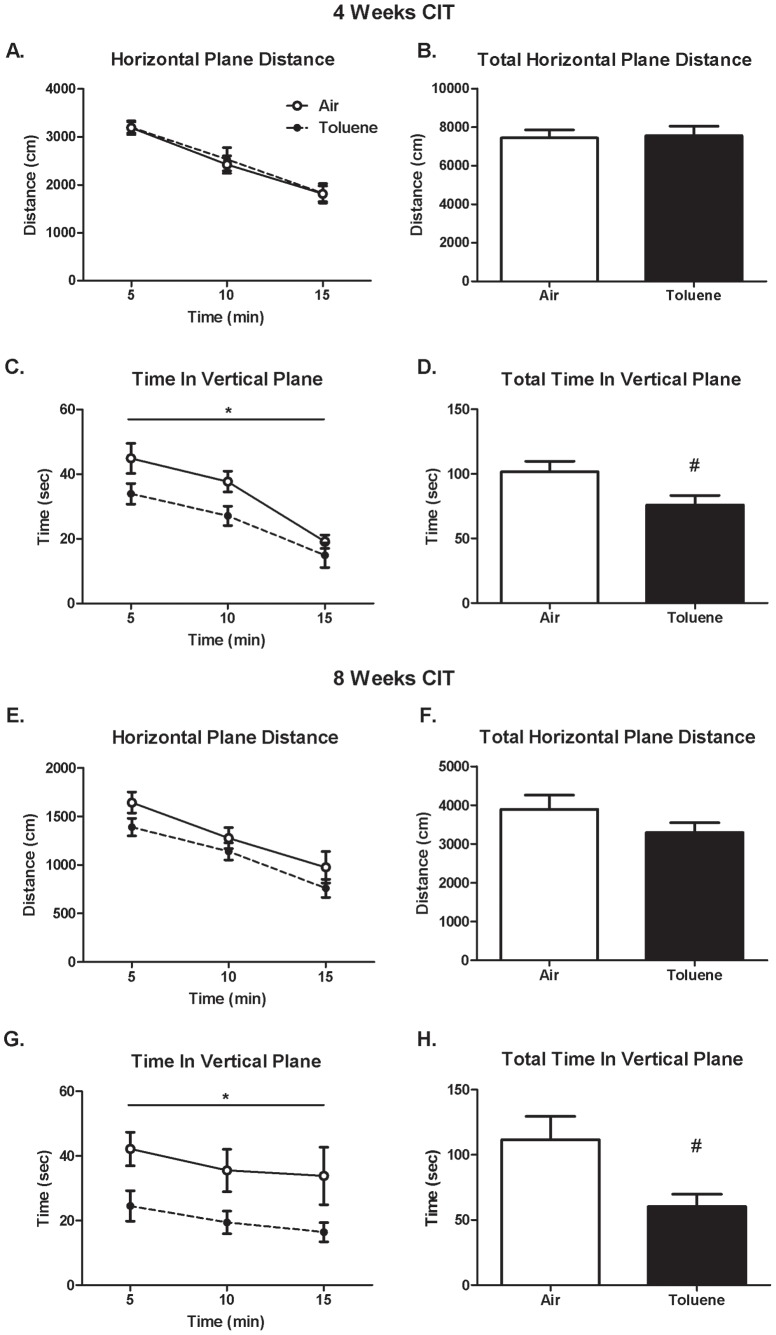
Basal locomotor activity. Basal locomotor activity following exposure to chronic intermittent toluene or air. Data are expressed as the mean (± SEM) distance moved in the horizontal plane (cm) measured in 5 minute time bins (A, E) or total distance (B, F) following 4 (A, B) or 8 (E, F) weeks exposure. Mean (± SEM) time spent in the vertical plane (cm) measured in 5 minute time bins (C, G) or total time spent in vertical plane (D, H) following 4 (C, D) or 8 (G, H) weeks exposure. *p<0.05 main effect of treatment (2-way RM ANOVA) or #p<0.03 (t-test), n = 12 per group at 4 weeks and n = 6 per group at 8 weeks.

Exposure to CIT during adolescence as well as into early adulthood had no effect on the distance travelled in the horizontal plane during a 15 minute session after either 4 (Figure 3A, B) or 8 (Figure 3E, F) weeks exposure compared to controls. There was a decrease in horizontal plane activity (main effect of time) in both air- and CIT-exposed rats over the 15 minute session at both 4 (F_(2,44)_ = 75.192, p<0.001, RM ANOVA) and 8 (F_(2,20)_ = 103.528, p<0.001, RM ANOVA) weeks. In contrast, CIT exposure for 4 weeks attenuated activity in the vertical plane (rearing) compared to controls (Figure 3C). A 2-way RM ANOVA revealed a main effect of treatment (F_(1,44)_ = 5.456, p = 0.029) and time (F_(2,44)_ = 36.049, p<0.001) on the time spent in the vertical plane, but no significant interaction between these factors (Figure 3C). A similar effect was observed for the number of entries into the vertical plane during this period (data not shown). Overall, CIT-exposed animals had a 26% reduction in the total time spent in the vertical plane (*t*-test, p = 0.028, Figure 3D) and a 22% reduction in the number of entries into the vertical plane (air: 79±7 vs CIT: 62±4 entries, p = 0.047, *t*-test) within the 15 minute session compared to air-exposed animals. After 8 weeks exposure to CIT in a separate cohort of animals, there was a more pronounced deficit in the time spent in the vertical plane (Figure 3G, H). A 2-way RM ANOVA revealed a main effect of treatment (F_(1,20)_ = 6.399, p = 0.030), but no main effect of time or interaction between these factors. A similar effect was observed for the number of entries into the vertical plane during this period (data not shown). Overall CIT-exposed animals had a 44% reduction in the total time spent in the vertical plane (*t*-test, p = 0.029, Figure 3H) and a 38% reduction in the number of entries into the vertical plane (air: 64±8 vs CIT: 40±5 entries, p = 0.024, *t*-test) over the 15 minute session when compared to air-exposed animals.

**Figure 4:**
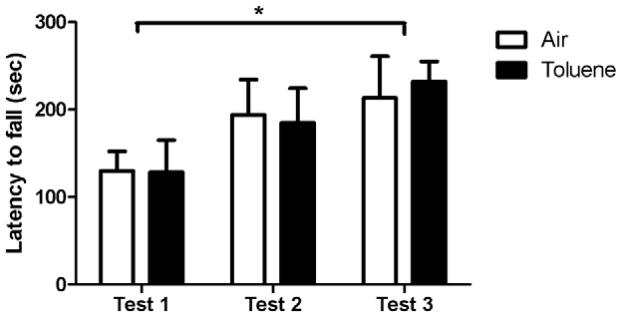
Rotarod. Latency to fall from the rotarod (sec) over the three test periods. Data are expressed as mean (± SEM). *p<0.01 test 1 verses test 3 for both air and chronic intermittent toluene-exposed animals (2-way RM ANOVA with Holm–Sidak post-hoc tests), n = 6 per group.

Altered vertical plane activity at 8 weeks could not be explained due to deficits in motor coordination as there was no difference in the latency (sec) to fall from the rotarod in any of the three test sessions between animals exposed to CIT compared to controls (Figure 4). A 2-way RM ANOVA revealed a main effect of time (F_(2,20)_ = 11.277, p<0.001), with post hoc analysis indicating that both groups displayed an increase in motor coordination as reflected by an increase in the latency to fall from the rotarod between the first and third test sessions (air p = 0.008, CIT p = 0.002, Figure 4). However, there was no main effect of treatment or interaction between factors.

### Effect of CIT exposure on MRI parameters

The region-based image analysis from T_2_-weighted images revealed no significant differences in the volume of the anterior commissure between CIT and air-exposed rats either prior to exposure (0 weeks) nor during the exposure period (4 and 8 weeks), or following 8 weeks abstinence (16 weeks) (Table 2). Nor was there a difference in the volume of the anterior commissure over this period irrespective of treatment group. Similarly in the genu, body and splenium of the corpus callosum there were no significant differences between groups at any time point measured (0, 4, 8 and 16 weeks, Table 2). A 2-way RM ANOVA revealed a significant increase in the volume of the corpus callosum (all regions) in both CIT and air-exposed animals over the 8 week exposure period; genu (F_(2,20)_ = 15.323, p<0.001; 0 vs 8 weeks *p*<0.001), body (F_(2,20)_ = 57.28, p<0.001; 0 vs 8 weeks *p*<0.001), splenium (F_(2,20)_ = 41.134, p<0.001; 0 vs 8 weeks *p*<0.001) (Table 2).

**Table 2:** Volume measurements of the anterior commissure and corpus callosum during the 8 week exposure period and following 8 weeks abstinence determined from T_2_-weighted MRI images.

Anterior Commissure Volume (mm^3^)			Corpus Callosum Volume (mm^3^)					
			Genu		Body		Splenium	
Time	Air (6)	CIT (6)	Air (6)	CIT (6)	Air (6)	CIT (6)	Air (6)	CIT (6)
0 Weeks	2.33±0.15	2.42±0.15	2.73±0.30	2.63±0.14	12.65±0.68	12.92±0.43	3.49±0.29	4.00±0.35
4 Weeks	2.75±0.35	2.73±0.37	3.78±0.15	2.27±0.11	17.45±1.05	18.07±1.02	5.85±0.34	5.29±0.25
8 Weeks	2.66±0.29	3.09±0.40	3.79±0.19†	3.82±0.23†	22.01±1.33†	20.59±0.61†	5.97±0.34†	5.81±0.16†
16 Weeks (8 Weeks Abstinence)	2.76±0.23	2.61±0.34	4.47±0.26	4.67±0.27	24.68±1.65	24.74±1.40	7.76±0.23	8.11±0.29

Data are expressed as mean of means (± SEM).

† Effect of time from 0 to 8 weeks (two-way RM ANOVA with Holm–Sidak post-tests). CIT: chronic intermittent toluene.

### Effect of CIT exposure on DTI parameters

**Figure 5:**
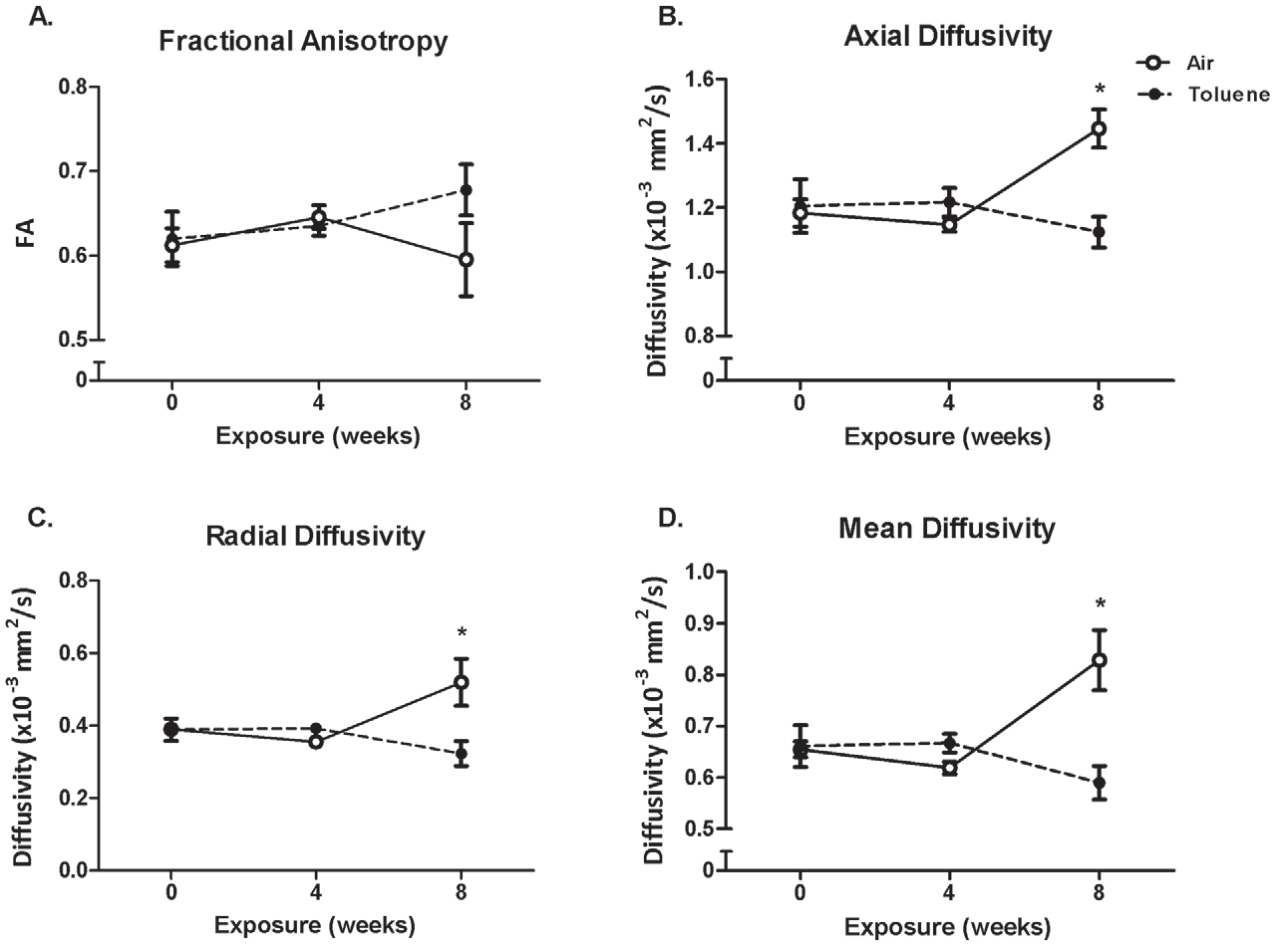
DTI parameters over the exposure period. DTI parameters including fractional anisotropy (A, FA), axial diffusivity (B), radial diffusivity (C) and mean diffusivity (D) in the anterior commissure during the 8 week exposure period to chronic intermittent toluene or air. There was a significant decrease in axial, radial and mean diffusivity at the 8 week time point in chronic intermittent toluene-exposed rats. Data are expressed as mean of means (± SEM). *p<0.05 difference between groups (2-way RM ANOVA with Holm–Sidak post-hoc tests). n = 6 per group.

**Figure 6:**
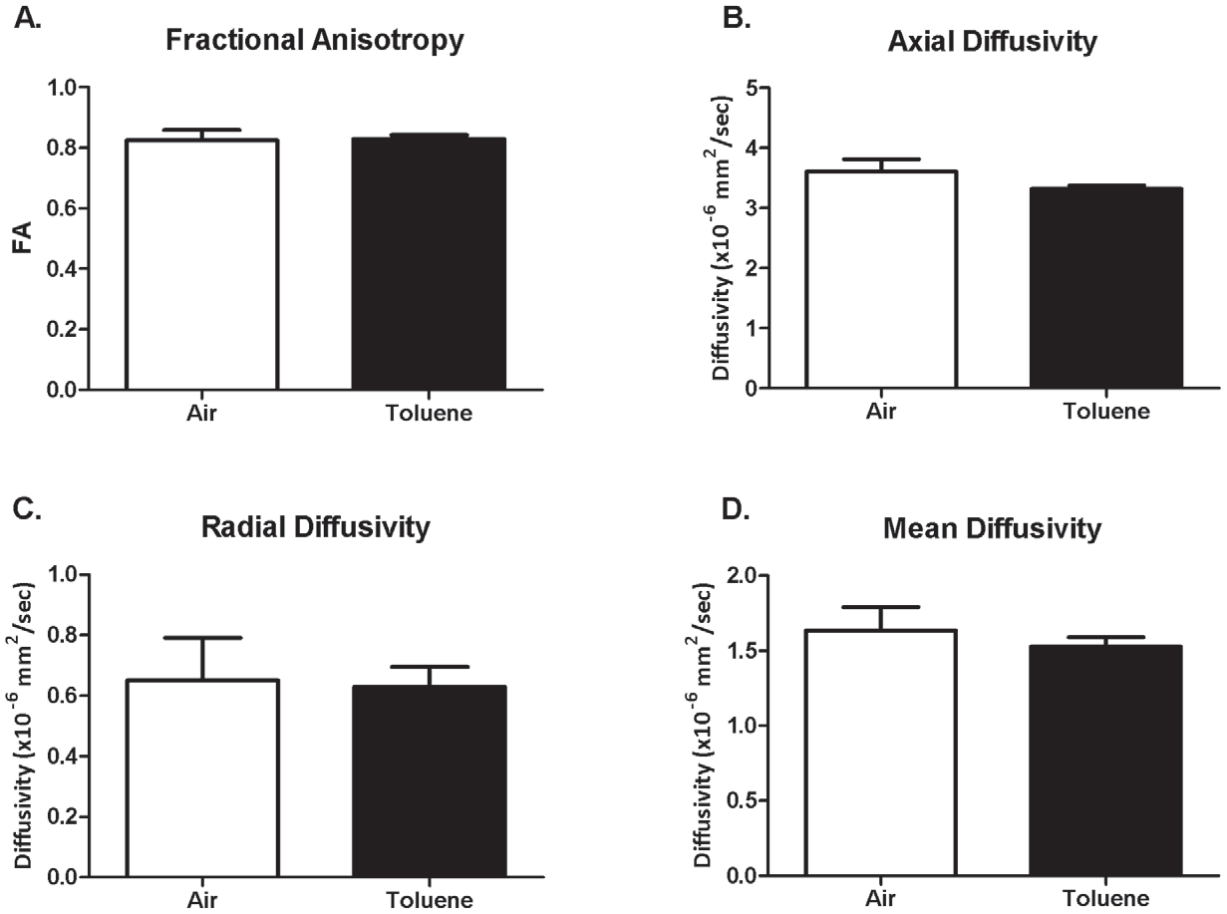
DTI parameters following abstinence. DTI parameters including fractional anisotropy (A), axial diffusivity (B), radial diffusivity (C) and mean diffusivity (D) in the anterior commissure after 8 weeks abstinence following exposure to chronic intermittent toluene or air for 8 weeks. Data are expressed as mean of means (± SEM). No difference between groups (*t*-test). n = 6 per group.

Region-based image analysis of DTI parameters (FA, D_A_, D_R_, D_M_) for the anterior commissure are summarized in Figure 5. Although CIT-exposed animals had increased FA values compared to controls after 8 weeks exposure, a 2-way RM ANOVA revealed no significant difference between groups. D_A_, D_R_ and D_M_ did not change over the first 4 weeks of the exposure period (irrespective of treatment), however these parameters increased from 4 to 8 weeks in air-exposed animals but not in those exposed to CIT. Consequently, a 2-way RM ANOVA revealed a significant interaction (time×treatment) for D_A_ (F_(2,18)_ = 5.671, p = 0.012), D_R_ (F_(2,18)_ = 7.398, p = 0.005) and D_M_ (F_(2,18)_ = 8.136, p = 0.003) over the exposure period, with post hoc analyses indicating a significant reduction in CIT-exposed animals compared to air-exposed animals at 8 weeks for all three parameters (p<0.001 in all cases). Following 8 weeks of abstinence, there were no differences between CIT and air-exposed animals on any of the parameters measured (Figure 6).

The region-based image analysis of DTI parameters for the corpus callosum are summarized in Table 3. Analysis using a 2-way RM ANOVA revealed no significant differences in any DTI parameter (FA, D_A_, D_R_, D_M_) over the 8 week exposure period, nor following 8 weeks recovery. There was a significant effect of time for FA values over the 8 week exposure period irrespective of treatment; genu (F_(2,18)_ = 4.856, p = 0.021), body (F_(2,14)_ = 4.187, p = 0.038), splenium (F_(2,14)_ = 8.788, p = 0.003), most likely reflecting normal maturational processes occurring in this region over adolescence and into early adulthood in rats [35], [36]. However, there was no effect of treatment or interaction between groups.

**Table 3:** DTI parameters including fractional anisotropy, axial diffusivity, radial diffusivity and mean diffusivity in the corpus callosum during the 8 week exposure period and following 8 weeks abstinence.

Corpus Callosum								
	0 Weeks		4 Weeks		8 Weeks		16 Weeks* (8 Weeks Abstinence)	
	Air (6)	CIT (6)	Air (6)	CIT (6)	Air (6)	CIT (6)	Air (6)	CIT (6)
Genu								
FA	0.568±0.020	0.551±0.018	0.610±0.018	0.614±0.026	0.530±0.017†	0.573±0.008†	0.693±0.034	0.777±0.035
D_A_	1.212±0.024	1.202±0.060	1.245±0.027	1.279±0.035	1.200±0.031	1.113±0.049	0.410±0.020	0.400±0.034
D_R_	0.443±0.017	0.463±0.039	0.416±0.024	0.428±0.035	0.502±0.017	0.425±0.024	0.115±0.010	0.0872±0.018
D_M_	0.700±0.010	0.709±0.045	0.692±0.021	0.711±0.031	0.734±0.016	0.650±0.032	0.071±0.003	0.064±0.007
Body								
FA	0.474±0.014	0.492±0.023	0.553±0.030	0.568±0.022	0.523±0.021†	0.527±0.009†	0.664±0.042	0.698±0.023
D_A_	1.159±0.033	1.153±0.065	1.197±0.019	1.205±0.009	1.189±0.048	1.089±0.039	0.393±0.019	0.386±0.016
D_R_	0.535±0.022	0.533±0.046	0.469±0.046	0.464±0.023	0.506±0.033	0.453±0.021	0.130±0.011	0.113±0.002
D_M_	0.742±0.024	0.740±0.052	0.715±0.033	0.711±0.014	0.740±0.036	0.679±0.025	0.073±0.004	0.679±0.002
Splenium								
FA	0.553±0.027	0.611±0.023	0.663±0.016	0.664±0.012	0.565±0.015†	0.561±0.013†	0.755±0.028	0.783±0.036
D_A_	1.250±0.066	1.342±0.070	1.428±0.0150	1.395±0.027	1.275±0.019	1.219±0.042	0.430±0.029	0.436±0.015
D_R_	0.474±0.029	0.437±0.042	0.429±0.018	0.430±0.009	0.498±0.022	0.466±0.032	0.097±0.012	0.088±0.014
D_M_	0.732±0.029	0.739±0.049	0.748±0.073	0.751±0.007	0.757±0.017	0.726±0.033	0.069±0.004	0.068±0.004

Fractional anisotropy (FA), axial diffusivity (D_A_, ×10^−3^ mm^2^/sec or ×10^−6^ mm^2^/sec*), radial diffusivity (D_R_, ×10^−3^ mm^2^/sec or ×10^−6^ mm^2^/sec*) and mean diffusivity (D_M_, ×10^−3^ mm^2^/sec or ×10^−6^ mm^2^/sec*). Data are expressed as mean of means (± SEM).

† Effect of time from 0 to 8 weeks (two-way RM ANOVA with Holm–Sidak post-tests).

* Note units at 16 weeks are different due to scans being performed *ex vivo*. CIT: chronic intermittent toluene.

## Discussion

In this study, we performed *in vivo* longitudinal MRI and DTI as a sensitive, reliable and non-invasive way to assess the structural integrity of white matter tracts following exposure to CIT during adolescence and early adulthood. Throughout the exposure period, we paired this with tests of basal locomotor activity and motor coordination. The results from this study demonstrate that exposure to CIT throughout adolescence and early adulthood in a paradigm that mimics human patterns of exposure, at clinically relevant concentrations, results in progressive deficits in vertical plane activity. In contrast, DTI detectable white matter abnormalities were only present following 8 weeks of exposure. Furthermore, these abnormalities were region-specific occurring in the anterior commissure but not the corpus callosum. There was however, apparent recovery of DTI detectable white matter abnormalities following a period of sustained abstinence.

In this study, we exposed rats to CIT at a time point equivalent to early adolescence (PND 27) to reflect the age at which many young people begin experimenting with inhalants [7], [37]. We used a concentration of toluene that acutely alters dopamine release in brain regions associated with drug reward [8], has been demonstrated to be positively reinforcing in both mice [29] and rats [31], and supports self-administration in primates [38]. While all rats displayed increased body weights during the exposure period, reflective of growth and development, in CIT-exposed animals this increase was significantly retarded after prolonged exposure (from 5 weeks on). A linear decrease in body weight has been found with increasing toluene concentrations when animals are exposed to toluene prenatally, which recovers over the first 2 weeks after birth [39]. In our study, differences in body weight were maintained for up to 12 days after the last exposure to CIT but recovered by 8 weeks following the last exposure. The effects on body weight were not reflected in either the weight of the brain or lungs and may be accounted for by altered body composition, nutrient absorption or metabolism [40]. Indeed human abusers often present as emaciated [18].

With respect to inhalant-induced neuropathology, human imaging studies consistently present T_2_-weighted hyperintensities of the deep cerebral, periventricular, cerebellar and internal capsule white matter in chronic inhalant abusers [14], [41], [42], which is thought to be indicative of demyelination [42]. Consistently presented neuropathology also includes loss of grey-white matter demarcation, thinning of the corpus callosum, atrophic dilation of the ventricles and cerebral atrophy. In the present study, we investigated the effects of CIT on white matter maturation as reflected by changes to DTI parameters with age following longitudinal scans prior to, and during exposure to toluene. These changes presented as a decrease in D_A_ and D_R_ (and consequently decreased D_M_) in the anterior commissure following 8 weeks CIT exposure, which occurred in the absence of changes to the volume of this tract. D_A_ reflects changes in the diffusion of water parallel to fibre bundles and consequently changes to this parameter may reflect changes in axonal integrity [22], [23]. Conversely, D_R_ reflects changes to the diffusion of water molecules perpendicular to fibre bundles and changes to this parameter are believed to reflect differences in myelination, though cell packing density, gliosis and inflammatory responses may all impact upon these measures [22], [24]. However, it should be noted that D_A_ and D_R_ are two separate identities and changes do not always occur in parallel [23], [24].

As changes to both D_A_ and D_R_ were observed following CIT it suggests both axons and myelin are being targeted. Indeed, similar to our observations, both D_A_ and D_R_ are reduced in the white matter with no corresponding change in FA following ischemic stroke in rats [43]. These changes correlate to the structural breakdown of axons and the compression of axoplasma due to swelling of the myelin sheath (but retention of myelin itself) and damage to oligodendrocytes, respectively [44]. Age may also influence these responses as human imaging studies following traumatic brain injury in adolescents report a reduction in D_R_ in the white matter, which is different to the profile observed in adults following similar insults (see [45]). Interestingly, in studies which have characterised pathological changes to DTI parameters, D_A_ is reduced during the initial phase of demyelination coinciding with axonal swellings, an increase in the number of non-phosphorylated axons and microglial activation, which occurs without the loss of axons themselves [46]. Based on the literature [23], [46], if active demyelination was occurring following exposure to CIT we would have expected D_R_ values to be increased relative to air-exposed animals. However, as D_R_ was reduced following CIT, this may suggest changes to parameters affecting white matter other than demyelination itself, such as changes to the orientation or crossing of fibres, or factors contributing to altered extracellular space such as altered oligodendrocyte, astrocyte or microglial cell density [44]. This is supported by the observations by Aydin *et al.* (2003) who used single-voxel MR spectroscopy to show that in the white matter of human inhalant abusers there is a reduction of N-acetyl aspartate and an increase in myo-inositol levels. As choline levels, a marker of demyelination, were unaltered this study supports the idea that axonal damage and gliosis predominantly contribute to inhalant-induced white matter pathologies [47]. Rosenberg *et al.* (1988) however, argue that toluene-induced white matter pathologies detectable by MRI are related to myelin toxicity, as axonal integrity was preserved and gliosis was minimal in their study of chronic toluene abusers [41]. Though a direct pathological cause is hard to determine based on the literature, we would hypothesise that in our model CIT exposure during adolescence and early adulthood results in axonal injury in the anterior commissure in conjunction with a decrease in axoplasma space due to swelling of white matter components (either cells or myelin sheaths). However, further investigation at the microscopic level is needed as changes to DTI parameters are not always preserved following injury [25]. Irrespectively, our data suggest that CIT does induce changes to maturational processes occurring in the anterior commissure and consequently this has the potential to affect the speed of information processing in this tract. Indeed, changes to DTI parameters have been reported in the anterior commissure in patients with schizophrenia [48] and following neonatal hypoxemic-ischemic encephalopathy [49].

Interestingly, in the anterior commissure, white matter abnormalities appeared sometime between 4 (PND 55) and 8 (PND 80) weeks exposure (i.e., late adolescence and early adulthood) suggesting extended exposure at this concentration of toluene is needed before DTI detectable white matter abnormalities appear. Indeed, Aydin and colleagues (2002) have shown a time dependent effect of inhalant abuse in humans with MRI detectable changes associated with periods of abuse greater than 4 years. The authors suggest this represents a cumulative toxic effect of toluene in this region [14], with greater impairment being seen with continued use [50]. In toluene abusers, T_2_-weighted hyperintensities originate in the periventricular region and progress to subcortical regions [14]. Aydin *et al.* (2002) suggest that this white matter pathology may initially be diffuse and regionally restricted and progress with further abuse corresponding to levels of high demyelination and axonal loss [14], with more severe abnormalities evident with an earlier onset of abuse [10]. In line with this, chronic petrol sniffers with a longer history of abuse show greater cognitive impairments associated with lead encephalopathy [15], [51]. This supports the hypothesis that there is a progression of white matter damage, which increases with further use and manifests as a progressive decline in cognitive function [12]. In our study, there was very little change in DTI parameters from PND 27 to PND 55 irrespective of exposure, with values in line with those reported previously for Wistar rats [36]. However, from PND 55 onwards (late adolescence), there was a dramatic increase in D_A_ and D_R_ in air-exposed animals with continued maturation, likely to be reflective of structural reorganization during this period. Thus, we are currently unable to conclude whether exposure to CIT results in injury *per se* or retardation in the normal maturational processes occurring in the anterior commissure during the exposure period. A reduction in D_A_ would support the hypothesis of brain injury [24] though apparent stabilization of D_R_ values from 4 to 8 weeks in CIT animals relative to controls would support retarded maturation. Interestingly, the appearance of changes to DTI parameters in the anterior commissure corresponded to the effects observed on the retardation in body weight in CIT-exposed animals.

In contrast to the changes observed in the anterior commissure, CIT exposure did not result in significant changes to MRI or DTI parameters in the corpus callosum even though callosal abnormalities have been reported in human adolescent inhalant users [10], and following exposure to other drugs, such as cocaine [52] and marijuana [53]. While there was a suggestion of changes (a non significant decrease in D_A_ and D_R_) in the genu following CIT we did not observe significant regional difference across the genu, body and splenium subsequent to CIT, even though regional vulnerabilities in the corpus callosum are reported following exposure to drugs such as cuprizone [46]. We also observed an increase in the volume of the corpus callosum over the eight week exposure period, in comparison to no change in volume of the anterior commissure. Our data are in line with those of Jito *et al.* (2008) and Bockhurst *et al.* (2008) who reported similar differences in maturational profiles of these regions [35], [36], which suggests that the corpus callosum and anterior commissure are undergoing different maturational profiles during the exposure period which may aid in the explanation of the regional vulnerabilities to CIT observed in this study.

Previous studies have quantified the impact of toluene inhalation on acute changes to behaviour, including locomotor activity, which appear biphasic and concentration-dependent [6], [54]. Lower concentrations of toluene, including the range used in the current study, induce motor excitation, while doses higher than 6,000 ppm induce motor impairment, sedation and anaesthesia [7]. Furthermore, the effects on locomotor activity appear age dependent with adolescent animals being more sensitive to the initial effects of toluene, at least to short exposures of high concentrations (8,000–16,000 ppm) [6]. Three days after the last exposure to CIT during adolescence and early adulthood we observed no difference in basal locomotor activity in the horizontal plane. However, the number of entries and time spent in the vertical plane during this period were significantly reduced. In rodents, vertical plane activity is reflective of rearing behaviour, changes in which are associated with environmental novelty and spontaneous exploration typically elucidating emotional as well as cognitive responses. This activity can be influenced by fear and anxiety [55], and has been attributed to non-specific excitability levels including motivation and arousal.

As changes in rearing occurred in the absence of changes in horizontal plane activity, the observed deficit is seemingly not due to a simple reduction in overall activity following CIT. Nor is it likely to be due to cerebellar dysfunction or defective motor learning as no differences were observed in the rotarod test, with both groups displaying increased motor coordination over time. Furthermore, deficits in vertical plane activity were more pronounced following 8 opposed to 4 weeks CIT exposure, an observation supported by human studies demonstrating greater cognitive and behavioural impairments in longer-term abusers [11]. Evidence suggests that ambulation (horizontal plane locomotor activity) and rearing (vertical plane activity) are not necessarily correlated, can be readily dissociated and that partially distinct physiological mechanisms are involved (see [56]). Consequently, horizontal locomotor activity is considered to be related to motor function whereas vertical plane activity is more related to cognitive functions. Thus, specific effects on vertical plane activity in this study may suggest that cognitive, as opposed to motor function may be more sensitive to the effects of CIT exposure during adolescence and early adulthood [57].

To our knowledge, the only other studies that have investigated the effects of toluene on rearing behaviour have looked at the effects following subchronic exposure at low concentrations, reflective of occupational exposure rather than abuse concentrations. Similar to our observations, Bergurger *et al.* (2003) found exposure to 40 ppm toluene for 12 hours a day, for 104 hours a week over 16 weeks, did not alter horizontal plane locomotor activity but decreased rearing activity when assessed one day after the last exposure [57], [58]. In contrast, Van Euler *et al.* (2000) found that chronic repeated exposure to 80 ppm toluene for 6 hours a day over 4 weeks led to a trend for increased rearing activity [59]. Rearing activity is also temporarily reduced following exposure to nicotine [60], but this also occurs in the presence of alterations to overall locomotor activity associated with decreased motivation and exploration.

Toluene's addictive properties, like other drugs of abuse, are attributed to its actions on dopamine within the mesocorticolimbic system. Toluene injected directly into the ventral tegmental area increases dopaminergic neuronal firing and dopamine release in the nucleus accumbens [8], with chronic exposure to toluene leading to persistent dopaminergic dysfunction [61]. While no one neural pathway appears linked to the control of rearing activity, the mesocorticolimbic system, including dopaminergic neurons, are necessary for normal locomotor and exploratory behaviour [9], [62], with limbic-striatal connections thought to play key roles in the initiation and maintenance of locomotor activity [63] and lesions of the ventral tegmental area being sufficient to decrease rearing activity [64]. In line with this, altered ventral striatal dopamine activity and medial frontal cortical serotonergic activity [65] has been observed in Wistar rats with low rearing activity in a novel open field, however rearing activity normalises once novelty is removed following repeated exposure [56]. Furthermore, injections of glutamate into the core of the nucleus accumbens reduce rearing without changes to overall locomotion [66], while infusion of the gamma-aminobutyric acid_B_ receptor agonist baclofen, is sufficient to inhibit amphetamine-induced increases in rearing activity [67]. Together, this suggests that several neurotransmitter systems influence behaviours mediating rearing activity, many if not all of which are affected by toluene [68], [69]. However, due to the diversity of toluene's effects upon the brain [70], direct interpretation of the changes in rearing activity following CIT exposure is difficult.

At present, it is unclear whether deficits in rearing activity following CIT exposure are related to the changes observed in the anterior commissure. The anterior commissure contains fibres from the olfactory bulb, temporal, orbitofrontal and pre-piriform cortices and the amygdala, thus connecting regions involved in cognitive processing, reward, decision making, memory and anxiety. Indeed, areas that continue to mature through adolescence are associated with fibres related to frontal pathways including those connecting the striatum and prefrontal regions [71]. However, the deficit in rearing appeared to be progressive, being present by 4 weeks (PND 55) after initiation of CIT exposure, with nearly a two fold increase in the observed deficit as exposure continued for up to 8 weeks (PND 80). This did not correlate with the appearance of changes to DTI parameters in the anterior commissure, which were not present by 4 weeks. This suggests that while DTI detectable white matter abnormalities may contribute to and/or escalate the deficit in rearing activity, they do not appear primarily responsible. Indeed, in humans a relatively short period of toluene exposure (8 months) results in impaired short-term memory and tactical performance [18]. This occurred despite no apparent abnormalities on T_1_ or T_2_ weighted MR images, suggesting a disconnect between toluene induced behavioural deficits (including cognitive function) and white matter pathology.

The potential for recovery of inhalant induced abnormalities is still debated. Cairney *et al.* (2005) demonstrated a relationship between the degree of neurobiological impairment and duration of abuse and that following 2 years abstinence, there was significant behavioural and cognitive recovery in all patients [15]. These data are supported by improvements in cerebral perfusion impairments in the frontal cortex and basal ganglia as well as clinical and neuropathological improvements following 14 months abstinence from chronic glue sniffing [18], and 6 weeks after chronic accidental solvent exposure [19]. However, Deleu and Hannssens (2000) showed no improvement in postural tremor following 5 months abstinence from chronic inhalant abuse [16] and Rosenberg *et al.* (1988) reported only 1 of 9 chronic toluene abusers showed consistently improved neurologic function with abstinence over a period of 18 months, yet this individual showed no change in toluene-induced white matter damage [17]. Furthermore, Dingwall *et al.* (2011), reported that the majority of deficits observed in complex cognitive tasks in chronic petrol sniffers normalised with 6 weeks abstinence, however deficits in aspects of learning and memory and executive functions persisted after 12 months abstinence suggesting a degree of permanency in some measures [72]. Despite our scans at 16 weeks being *ex vivo*, white matter abnormalities were no longer apparent in CIT-exposed animals compared to air controls following an extended period of abstinence. This was supported by normalization of toluene induced deficits in body weight. Thus, the observations of reduced D_A_ and D_R_ in our study would support acute changes to white matter parameters, and increases the possibility of reversal of MRI/DTI detectable white matter pathology over time.

### Conclusions

CIT exposure during adolescence and early adulthood results in distinct patterns of white matter abnormalities in rats. These abnormalities appear to be region-specific and may, in part, be attributed to the different maturational states of individual white matter pathways during the exposure period. Furthermore, the appearance of CIT-induced white matter abnormalities was time dependent, occurring between 4 and 8 weeks after the initiation of the toluene exposure. In contrast, we observed a progressive decrease in rearing activity but no change in ambulation, which was present by 4 weeks exposure to CIT, suggesting that toluene may have an effect on behaviour prior to the appearance of DTI detectable abnormalities in the brain, at least in the parameters measured in this study. This suggests different mechanisms sensitive to CIT-induced changes may be involved in the modulation of rearing activity other than injury to white matter pathways. Reversal of DTI detectable microstructural white matter abnormalities following a period of abstinence supports the potential for recovery.
